# Characterization of heterogeneous vancomycin-intermediate resistance, MIC and accessory gene regulator (*agr*) dysfunction among clinical bloodstream isolates of *staphyloccocus aureus*

**DOI:** 10.1186/1471-2334-11-287

**Published:** 2011-10-25

**Authors:** Yoriko Harigaya, Dung Ngo, Alan J Lesse, Vanthida Huang, Brian T Tsuji

**Affiliations:** 1 Laboratory for Antimicrobial Pharmacodynamics, School of Pharmacy and Pharmaceutical Sciences and The New York State Center of Excellence in Bioinformatics & Life Sciences, University at Buffalo, State University of New York, New York, USA; 2 School of Medicine and Biomedical Sciences, University at Buffalo, State University of New York, New York, USA; 3 Mercer University, College of Pharmacy, Atlanta, Georgia, USA; 4 Roswell Park Cancer Institute Departments of Medicine, Buffalo, New York, USA; 5 Food and Drug Administration, Center for Drug Evaluation and Research, Silver Spring, Maryland, USA

## Abstract

**Background:**

The development of hVISA has been associated with vancomycin clinical failures and is commonly misidentified in clinical microbiology laboratories. Therefore, the objectives of this present study was to improve the reliability of methodologies and criteria for identifying hVISA, evaluate the prevalence of hVISA among clinical bloodstream isolates of *S. aureus *and determine if there exists a relationship between accessory gene regulator (*agr) *dysfunction and the hVISA phenotype.

**Methods:**

The presence of hVISA in 220 clinical *S. aureus *isolates (121 MSSA, 99 MRSA) from bloodstream infections was examined by CLSI broth microdilution, Macro & Standard Etest. Isolates which were classified as hVISA by Macro Etest, were additionally evaluated using a modified PAP-AUC method using a modified starting inoculum of 10^10 ^CFU/mL, and growth on brain heart infusion agar with 4 mg/L vancomycin (BHIV4) at 10^8 ^and 10^10 ^CFU/mL, and *agr *function was assessed by delta-hemolysin production.

**Results:**

Broth microdilution MIC_50/90 _of *S.aureus *and hVISA was 1.0/2.0 and 1.5/2.0 mg/L (*p*= 0.02), respectively. Macro Etest identified 12 (5.5%) hVISA isolates; higher among MRSA (9.1%) versus MSSA (2.5%) (*p *= 0.03). The mean modified PAP-AUC ratios (> 0.8) of 7 MRSA strains and 3 MSSA strains were significantly different (*p *= 0.001). 58% of hVISA strains were found to be *agr *dysfunctional when 21% of MRSA strains were *agr *dysfunctional. hVISA was detected among *S. aureus *bloodstream isolates, which were classified as susceptible among clinical microbiology laboratories.

**Conclusions:**

Evaluating the correlation between Etest MICs and modified PAP-AUC ratio values will add further improvement of discriminating hVISA, and *agr *dysfunction may be predictive of strains which display a greater predilection to display the hVISA phenotype.

## Background

Heteroresistant vancomycin-intermediate *Staphylococcus aureus *(hVISA) has been detected in clinical *S. aureus *strains throughout the world [1-3] and has been associated with vancomycin treatment failure for *S. aureus *[4]. The prevalence of hVISA has been reported ranging from 0 to 74% [2,5]. The large variance of the hVISA prevalence is due to the lack of a prevalence study. On top of that, standardized methodologies and criteria to detect hVISA in the clinical microbiology laboratory are not established. The prevalence study with high quality cost effective methodologies are necessary to be conducted with uniformly to identify the true hVISA prevalence. Owing to the unstable characteristics of vancomycin heteroresistant phenotype, establishment of standardized methods to detect hVISA has been challenging [5].

hVISA was first characterized in 1997 [6]. hVISA is defined as isolates which are classified by the Clinical and Laboratory Standard Institute (CLSI) as having a vancomycin broth microdilution minimum inhibitory concentration (MIC) in the susceptible range where a notable subpopulation of cells (approximately one organism in every 10^5^-10^6 ^or more) with MICs over the susceptibility breakpoint [6]. A number of current methodologies for detection of hVISA exist such as the Etest Macromethod, Etest Glycopeptide Resistance Detection (Etest GRD: Standard Etest) method and the population analysis profile-area under the curve (PAP-AUC) method [7]. Multiple studies demonstrated high sensitivity, high specificity, consistent and low variation between laboratories in detecting hVISA by the Macro Etest Method [8-10]. Although PAP-AUC is labor intensive, it has long been considered the gold standard for hVISA detection [2]. Furthermore, as it relates to genetic factors which can be markers for hVISA, no single resistance gene has been correlated with hVISA development [11,12]; however, it has been demonstrated that the expression of hVISA phenotype is well associated with dysfunction in the quorum sensing cluster of genes, the accessory gene regulator group (*agr*), controlling colonization and virulence, as well as a number of other regulators [13].

In this study, the objectives were to: 1) determine the prevalence of hVISA in clinical *S. aureus *bloodstream isolates utilizing Macro Etest, Standard Etest, modified PAP-AUC method and Brain Heart Infusion Agar containing vancomycin 4 mg/L (BHIAV4) method. 2) determine the relationship between hVISA, vancomycin MIC, and *agr *dysfunction among clinical *S. aureus *bloodstream isolates for the purpose of establishing the standardized method to facilitate the hVISA detection in the clinical setting.

## Methods

### Bacterial Isolates

220 clinical *S. aureus *isolates obtained from the blood of infected patients at the Buffalo Veterans Affairs Health System of Western New York from 2003 to 2005 were analyzed. Isolates were obtained from 220 different patients. All studies were conducted in accordance with the Institutional review board at the University at Buffalo and the VA Health System. Control MSSA strain *S. aureus *ATCC29213 and a standard hVISA strain Mu3 (ATCC700698) were utilized.

### Antimicrobial agents, Media, and MIC Determination

Vancomycin powder (Sigma Chemical Co, St. Louis, MO) was obtained commercially. Stock solutions were made according to manufacturer's directions and stored at 4°C. Etest strips of vancomycin (AB Biodisk, Solna, Sweden) were utilized for the initial screening of hVISA. All susceptibility testing used Mueller-Hinton Broth (Difco Laboratories, Detroit, MI) supplemented with calcium (25 mg/L) and magnesium (12.5 mg/L). Brain-Heart Infusion agar (Difco Laboratories, Detroit, MI) was utilized for all analysis. Trypticase soy agar with 5% sheep blood agar (TSA II, Becton-Dickinson Diagnostics, Sparks, MD) was utilized for a bacteria growth medium and delta-hemolysin test. CLSI Broth microdilution MICs were determined for 220 clinical isolates and a control MSSA strain *S. aureus *ATCC29213 and a standard hVISA strain Mu3 in triplicate accordance with the Clinical and Laboratory Standards Institute (formerly the National Committee for Clinical Laboratory Standards) guidelines [14].

### Macro and Standard Etest

The detection of hVISA among 220 isolates was completed by using the Etest Macro Method as a preliminary screen as previously described [7,15]. Overnight growth cultures of the 220 clinical isolates and a control MSSA strain *S. aureus *ATCC29213 and a standard hVISA strain Mu3 were suspended in saline solution. The suspensions were adjusted to 2.0 McFarland, swabbed on the BHI plates and dried at room temperature, and Etest strips were placed on the plates. After 48 h incubation at 35°C, the intersections of the elliptical inhibition zone and the subpopulation growth in the inhibition zone were recorded. The criterion for Macro Etest for detecting hVISA was the appearance of one or more subpopulation colonies in MIC ≥4 mg/L refers to the recent study results performed by Maor [16]. Additionally, Standard Etest was performed at a 0.5 McFarland using MHA that was also performed in the similar manner and interpreted according to CLSI breakpoints.

### Modified PAP-AUC

Isolates which displayed hVISA profile by the Macro Etest Method were further analyzed using modified PAP-AUC method according to the standard PAP-AUC methodology as previously described [7]. Briefly, overnight growth culture of hVISA clinical isolates and a control isolates *S. aureus *ATCC29213 and a standard hVISA strain Mu3 were suspended in saline solution. The suspensions were adjusted to the modified starting inoculum 10^10 ^CFU/mL. Cultures were serially diluted from 0 to 10^-6^, and 10 μL of each dilution was plated in quadruplicate on BHI containing vancomycin in the following concentrations: 0.5, 1, 2, 4, 6, 8 and 16 mg/L. Colonies were enumerated after 48 h incubation at 35°C. Bacterial colony counts (Log_10 _CFU/mL) were plotted against the vancomycin concentration (0 to 4 mg/L) using SigmaPlot 9.0. The area under the curve (AUC) was calculated for each isolate and divided by the AUC value of the reference strain Mu3. The criteria for identifying hVISA were either the modified PAP-AUC ratio of > 0.9 or > 0.8. Additionally, modified PAP-AUC ratios were plotted against Macro Etest MICs, which were evaluated for the relation between two variables using the linear regression equation and the correlation coefficient (r).

### BHIAV4

To evaluate the inoculum effect of hVISA strains detected by the Macro Etest Method, a modified procedure involving Brain Heart Infusion agar contain vancomycin 4 mg/L BHIAV4 method was performed as described previously [6]. Briefly, overnight growth culture of hVISA clinical isolates and a control isolates *S. aureus *ATCC29213 and a standard hVISA strain Mu3 were suspended in saline solution. The suspensions were adjusted to the modified inoculum of 10^8 ^and an additional higher inoculum of 10^10 ^CFU/mL. Each sample was plated in quadruplicate on BHI agar containing vancomycin 4 mg/mL. Colonies were enumerated after 48 h incubation at 35°C. Growth of 1 or more colonies indicate a positive result.

### Delta-hemolysin

To evaluate the relationship between *agr *dysfunction and hVISA, delta-hemolysin expression was determined for hVISA isolates detected by Etest according to the study previously described [13]. Briefly, overnight growth culture of hVISA clinical isolates and *S. aureus *RN4220 were suspended in saline solution. The suspensions were adjusted to 0.5 McFarland standards and were streaked vertically near RN4220 on TSA II. After incubating plates at 35°C for 24 h, the expression of delta-hemolysin was evaluated.

### Statistical analysis

The relation between MICs (mg/L) values and modified PAP-AUC ratios was evaluated using the linear regression analysis. The correlation coefficient (r) was calculated to display the correlation between the two variables. The relationships between the independent and dependent variables were analyzed using Mann-Whitney U test and chi-square test. P-value of < 0.05 was considered statistically significant. The number of false positive, false negative, the percentage of sensitivity and specificity were calculated for each method.

## Results

**Figure 1:**
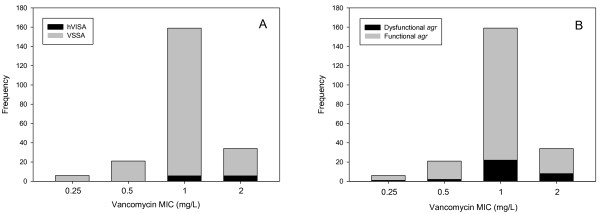
**MIC distribution of 220 *S. aureus *bloodstream isolates in relation to frequency of isolates that were hVISA (A) or *agr*-dysfunction (B)**.

The proportion of methicillin susceptible *S. aureus *(MSSA) and MRSA isolates in tested *S. aureus *isolates were 55 and 45%, respectively (Table 1). The CLSI broth microdilution MIC range for *S. aureus *was 0.25 to 2 mg/L, and for hVISA was 1 to 2 mg/L. The distribution of MICs 0.25, 0.5, 1 and 2 mg/L measured by the broth microdilution method were 6 (2.7%), 21 (9.5%), 159 (72.3%) and 34 (15.5%), respectively (Figure 1). MIC_50 _of *S. aureus *(n = 220), MSSA (n = 121), MRSA (n = 99) and hVISA (determined by Macro Etest, n = 12) were 1, 1, 1 and 1.5 mg/L, respectively. MIC_50/90 _of hVISA analyzed by broth microdilution, Standard Etest, and Macro Etest were 1.5/2, 4/4, and 6/8 mg/L, respectively.

**Table 1:** Summary of the prevalence of MRSA and hVISA in 220 *S.aureus *bloodstream isolates

	*S. aureus* 220	MSSA 55% (121/220)	MRSA 45% (99/220)	hVISA 5.5% (12/220)
MIC50/90	1/2	1/2 (*p *= 0.51)^a^	1/2 (*p *= 0.46)^a^	1.5/2 (*p *= 0.03)^a^
*agr*-dysfunction	15% (33/220)	10% (12/121)	21% (21/99) (*p *= 0.02)^b^	58% (7/12) (*p*< 0.0005)^c^

MIC_50/90 _determined by CLSI broth microdilution and the prevalence of agr-dysfunctional

^a ^MIC distribution of total clinical *S.aureus *versus MIC distribution of each phenotype (Mann-Whitney U)

^b ^Prevalence of *agr *dysfunction in MRSA isolates versus MSSA isolates (Chi-square test)

^c ^Prevalence of *agr *dysfunction in hVISA isolates versus VSSA isolates (Chi-square test)

**Figure 2:**
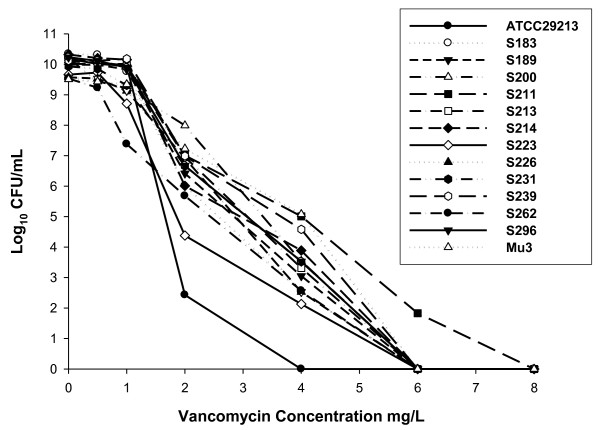
**Population analysis profiles of twelve hVISA clinical isolates, a control isolates *S. aureus *ATCC29213 and a standard hVISA strain Mu3**.

All tested *S. aureus *isolates displayed CLSI broth microdilution MIC values in the susceptible range, however, the distribution of MICs was significantly different between the total clinical *S. aureus *and hVISA strains (*p *= 0.02). Standard Etest applying the CLSI breakpoints identified 13 clinical *S. aureus *strains as hVISA, and Macro Etest classified 1 out of those 13 hVISA strains as a vancomcyin susceptible *S. aureus *(VSSA) which also displayed the low modified PAP-AUC ratio (0.54), as shown in PAP profiles in Figure 2. The criterion for Macro Etest for detecting hVISA was the appearance of one or more subpopulation colonies in MIC ≥4 mg/L. hVISA prevalence was higher in MRSA (9.1%) compared to MSSA (2.5%) (*p *= 0.03) (Table 2). The Etest MIC results displayed high reproducibility (SD < 10%).

**Table 2:** Summary of the shift in MIC and PAP-AUC ratio of 13 hVISA isolates screened by Standard Etest and a control *S. aureus *ATCC29213.

ID	Organism	BMD^1^	Standard Etest	Macro Etest	PAP-AUC Ratio	hVISA^2 ^	*agr*-function
S183	MSSA	2	3	6	0.83	Y	Positive
S189	MSSA	2	4	6	0.87	Y	Negative
S200	MRSA	1	3	6	0.95	Y	Negative
S211	MRSA	1	4	8	1.12	Y	Positive
S213	MRSA	1	4	8	0.91	Y	Positive
S214	MRSA	2	6	6	0.91	Y	Positive
S223	MRSA	2	4	6	0.7	Y	Positive
S226	MSSA	2	3	6	0.84	Y	Negative
S231	MRSA	1	4	6	0.9	Y	Negative
S239	MRSA	2	4	8	0.99	Y	Negative
S262	MRSA	1	3	4	0.75	Y	Positive
S285	MRSA	1	3	3	0.54	N	Negative
S296	MRSA	1	3	6	0.91	Y	Negative
ATCC29213	MSSA	1	2	3	0.53	N	Positive

^1^BMD: Broth Microdilution

^2^Identified by Macro Etest

With ten hVISA strains screened by Macro Etest and confirmed by modified PAP-AUC with the ratio cutoff value > 0.8, the mean modified PAP-AUC ratio (± SD) was (0.91 ± 0.06). Additionally, the mean modified PAP-AUC ratio (± SD) of seven hVISA isolates with MRSA phenotype was (0.94 ± 0.04), which was higher compared to the mean ratio of three hVISA isolates with MSSA phenotype (0.85 ± 0.02) (*p *= 0.001). With a higher endpoint criterion modified PAP-AUC ratio > 0.9, five hVISA strains were misclassified as susceptible. The percentage of the sensitivity modified PAP-AUC ratio > 0.8 and ratio > 0.9 in discriminating hVISA were 83.3% and 58.3%, respectively, when the sensitivity of broth microdilution, Standard Etest, BHIAV4 with lower inoculum (10^8 ^CUF/mL) and BHIAV4 with higher inoculum (10^10 ^CFU/mL) were 0%, 100%, 8.3% and 100%, respectively.

**Figure 3:**
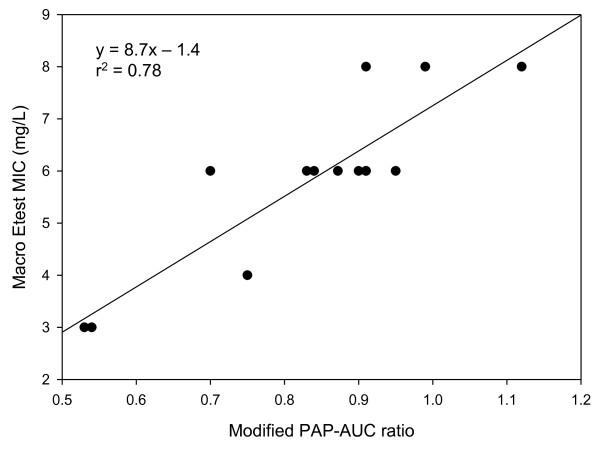
**Scatter plot displaying the correlation between Macro Etest MICs and modified PAP-AUC ratio of thirteen *S. aureus *clinical isolates identified as hVISA by Standard Etest and control isolate ATCC29213**.

Macro Etest MICs were plotted against modified PAP-AUC ratios (Figure 3). The regression line for the data of thirteen isolates identified as hVISA by Standard Etest is displaying the strong relationship between Macro Etest MIC shift and modified PAP-AUC ratio (r^2 ^= 0.78). Standard Etest also displayed the relationship between MIC values and modified PAP-AUC ratio (r^2 ^= 0.24). In contrast, the CLSI broth microdilusiton MIC values did not have clear correlation with modified PAP-AUC ratios (r^2 ^= 0.026). According to the linear regression equation for these two variables, Macro Etest MICs and modified PAP-AUC ratio, the estimated modified PAP-AUC ratio at MIC 4 and 6 mg/L were 0.62 and 0.85, respectively.

The percentages of *agr *dysfunctional phenotype in total tested *S.aureus*, MSSA, MRSA and hVISA were 15%, 10%, 21% and 58% (Table 1). The prevalence of *agr *dysfunctional phenotype was significantly higher in hVISA compared with VSSA (*p *= 0.02). Additionally, the prevalence of *agr *dysfunctional phenotype was higher in MRSA compared with MSSA (*p *= 0.02). BHIAV4 method analysis using lower inoculum (10^8 ^CFU/mL) misclassified 11 out of 12 hVISA strains as VSSA. On the contrary, BHIAV4 analysis with higher inoculum (10^10 ^CFU/mL) identified all 12 hVISA strains as hVISA.

## Discussion

Due to the heteroresistant nature of vancomycin intermediate resistance, a number of clinical *S. aureus *may exhibit resistant subpopulations not expressed by traditional methods of MIC detection. Therefore, it is necessary to investigate a variety of methods to increase sensitivity of hVISA detection in clinical laboratories. A recent multicenter, multi-national comparison by Wootton et. al. determined that the Macro Etest method displayed high sensitivity (85.9%) in detection of hVISA which is rationale of using this method as an initial screen for strains which display hVISA [9]. In the current study we utilized this methodology to determine that the hVISA phenotype was nearly four times higher among MRSA vs. MSSA. Quantitatively, these results were also confirmed by population analysis profiling methods whereby MRSA displayed the higher modified PAP-AUC ratios compared to hVISA MSSA.

This perhaps is intuitive due to vancomycin being commonly utilized for MRSA; however, hVISA was also detected among MSSA, which is in agreement with the recent findings of a significant increase in vancomycin MICs among MSSA clinical isolates by Wang [17]. These findings may suggest that the use of aggressive vancomycin regimens, in MRSA infections are important, so that the development hVISA phenotype which is driven by suboptimal vancomycin therapeutic exposure can be avoided. Certainly, alternative treatment should be used for MSSA infections in lieu of vancomycin treatment.

The BHIAV4 method described by Hiramatsu [6] has been evaluated in a number of studies. One concern with this methodology is that, the heteroresistant subpopulations arise in very low frequency, which results inability of the BHIAV4 method to detect heteroresistant phenotypes when the inoculum is low. To study the impact of inoculums on detection and mutation frequency, we utilized a high inoculum of *S. aureus *(10^10 ^CFU/ml) which is often observed in the severe *S. aureus *infection including infective endocarditis. As a result, the inoculum level displayed the significant impact on the sensitivity: the modified BHIAV4 method using the higher inoculum 10^10 ^CUF/mL 100% isolates additional hVISA correctly. Furthermore, at high bacterial density, stationary-phase growth foster biofilm formation via quorum-sensing mechanisms involving *agr *in *S. aureus *[13]. Coupled with the finding that proportion of *agr *dysfunctional strains were nearly five times higher among hVISA vs. VSSA, these findings may provide additional potential mechanistic insights into the development vancomycin resistance. Furthermore, *agr *dysfunctional *S. aureus *strains tend to display low activity in metabolic pathway which is growth phase dependant. Therefore, in the context of high bacterial density, it has been hypothesized that stationary phase growth, defective autolysis profiles, biofilim production and the production of thicker cell walls, may be facilitated by quorum sensing mechanisms [11-13]. Finally, from a clinical microbiology standpoint, the evaluation of delta-hemolysin production may be of potential utility to detect staphylococci which display an increase proclivity to develop heterogeneous resistance.

This study had potential limitations. First, we acknowledge the lack of geographically diverse sites for *S. aureus*, as isolates were from a single institution and limited to nosocomial bacteremia in a defined geographic area. Additional multicenter studies including both nosocomial and community-onset bloodstream infections are necessary to define the true prevalence of hVISA. Second, we utilized PAP using a high initial inoculum of 10^10 ^CFU/mL to confirm all hVISA isolates determined by the Etest Macro Method. Although, this method may decrease specificity, our VSSA control strain, ATCC 29213 did not display any vancomycin intermediate populations. Third, although we utilized delta-hemolysin expression as a surrogate marker measure of *agr *function, more quantitative measure of RNAIII expression may be necessary to further define the relationship *agr *and heteroresistance. Overall, the prevalence of hVISA phenotype in 220 clinical *S. aureus *isolates was observed to be 5.5% in *S. aureus *and 9.1% in MRSA strains using the Etest Macro Method, which was well correlated with modified PAP-AUC ratio values. *agr *dysfunction may add further utility to hVISA detection in the clinical microbiology setting.

## Conclusion

hVISA was detected among *S. aureus *bloodstream isolates, which were classified as susceptible among clinical microbiology laboratories. Evaluating the correlation between Etest MICs and modified PAP-AUC ratio values will add further improvement of discriminating hVISA, and *agr *dysfunction may be predictive of strains which display a greater predilection to display the hVISA phenotype.

## Competing interests

The authors declare that they have no competing interests.

## Authors' contributions

YH was responsible for overall study design, participated in the experimental work, conducted an extensive literature review, and wrote the manuscript. DN carried out the experimental work and participated in study design. VH participated in the study design and contributed to writing of the manuscript. AJL contributed ideas and characterization of strains. BTT was responsible for overall study design, conducted an extensive literature review, and wrote the manuscript. All authors have read and approved the final manuscript.

## Pre-publication history

The pre-publication history for this paper can be accessed here:

http://www.biomedcentral.com/1471-2334/11/287/prepub
